# Optimization of a Rhabdomyolysis Model in Mice With Exertional Heat Stroke Mouse Model of EHS-Rhabdomyolysis

**DOI:** 10.3389/fphys.2020.00642

**Published:** 2020-06-16

**Authors:** Si-Xiao He, Ru Li, Huo-Hong Yang, Zi-Qing Wang, Yan-Mei Peng, Jun-Hao Huang, Qiang Ma

**Affiliations:** ^1^Department of Biopharmaceutics, School of Laboratory Medicine and Biotechnology, Southern Medical University, Guangzhou, China; ^2^Cancer Research Institute, Southern Medical University, Guangzhou, China; ^3^Department of General Surgery, Nanfang Hospital, Southern Medical University, Guangzhou, China

**Keywords:** exertional heat stroke, classical heat stroke, rhabdomyolysis, creatine kinase, calcium overload

## Abstract

Exertional heat stroke (EHS) is a life-threatening disease characterized by high mortality and incidence of rhabdomyolysis (RM). It would therefore be valuable to establish a stable EHS-induced RM model that accurately reflects the clinical characteristics of EHS patients and provides an objective animal model for further study of the pathogenesis of RM. In the current study, 8∼9-week-old, male, wild-type C57BL/6J mice, at the stage of sexual maturity, were randomly divided into four groups: the EHS group, the classical heat stroke (CHS) group, the sham heat exercise group, and sham heat rest group. The survival rate of mice was determined under relatively high levels of temperature and humidity (37.5°C, 65% relative humidity (RH); 37.5°C, 70% RH; 39.5°C, 65% RH; and 39.5°C, 70% RH) as well as a high core temperature (Tc; 42, 42.5, and 43°C). Results showed that the environmental condition of 39.5°C and 65% RH was most suitable for EHS modeling. The end point of EHS evaluation was exhaustion or an individual’s core temperature reaching 43°C. The survival rate of mice in the EHS group within 24 h under these conditions was 37.34%, which is consistent with the high mortality characteristics noted in EHS patients. Severe RM was observed in the EHS group by H&E staining and transmission electron microscopy. Creatine kinase levels in the EHS group mostly exceeded 10,000 U/L, which was approximately 10 times higher than that in the sham heat rest group. Renal tubules of the EHS group exhibited severe necrosis, and calcium overload in the skeletal muscles of this group was also observed using intravital 2-photon microscopy. In conclusion, we made improvements to a stable EHS-induced RM animal model to truly reflect the clinical characteristics of EHS patients. This new model should be helpful in the further study of RM pathogenesis.

## Introduction

Exertional heat stroke (EHS) is the most complicated and dangerous form of heat stroke as it can lead to irreversible injury and even death (Bouchama and Knochel, 2002). It is a life-threatening condition that is characterized by an increase in core body temperature to >40°C and rhabdomyolysis (RM) (Casa et al., 2012).

One of the hallmarks of a good preclinical animal model for EHS is the ability to simulate the conditions underlying EHS to offer an understanding its pathogenesis. There are several EHS models, particularly those described by King et al. (King et al., 2015, 2017) or Hubbard et al. (1977, 1979) and Hubbard (1979). However, the existing models have limitations in that they cannot fully reflect the key clinical characteristics of EHS patients. Almost 99% of mouse genes are shared with the human genome (Dutta and Sengupta, 2016); thus, mouse models may be more suitable than rat models for studying EHS. Leon et al. have reported a mouse EHS model in which creatinine kinase (CK) values were increased but not significant; in addition, all the mice survived the 4 day trial; hence, there was no significant difference between the EHS group and the control group in terms of mortality (King et al., 2015, 2017). EHS is an acute injury associated with high morbidity and mortality, and is commonly encountered within the military, athletes, and construction workers (Adams et al., 2012). Of all the heat stroke cases in the U.S. Army between 1980 and 2002, 25% cases were associated with rhabdomyolysis, and 13% were associated with acute renal failure; mortality of EHS with hypotension was >30%. Other reports indicate that the EHS mortality rate over the last 50 years has remained between 10 and 50% at St Louis and Kansas City (Bouchama, 1995). Although various strategies for the treatment of EHS have been used over the past several decades, the clinical efficacy of therapeutic interventions is still limited. EHS currently continues to be a problem during military training and operations (Carter et al., 2005). With the high mortality rate and high incidence of RM in EHS patients, it is suggested that existing EHS animal models do not sufficiently encapsulate key clinical characteristics of the condition.

In the current study, we optimized the existing EHS modeling system which was mainly based on Leon’s reports (King et al., 2015, 2017) and established an EHS-induced RM mouse model that was found to be consistent with the clinical characteristics of EHS patients.

## Materials and Methods

### Animals

Because estrogen is known to affect heat tolerance and heat stroke-related organ injury (Chen et al., 2006), only male animals were used in the present study. Previous models of EHS have examined mice at the relatively late adult age of 4 months (King et al., 2015, 2017). In the current study, adult specific-pathogen-free C57BL/6 wild type mice, approximately 8∼9 weeks of age at the stage of sexual maturity, were purchased from Southern Medical University Animal Center (Guangzhou, China). The mice (four per cage) were housed under controlled environmental conditions [12 h light/dark cycle, 55 ± 5% relative humidity (RH), 23°C ambient temperature (Ta)] with free access to standard chow and water. All experimental protocols involving animals were approved by the Animal Care and Use Committee of the Southern Medical University in accordance with the Guide for the Care and Use of Laboratory Animals of the National Institutes of Health.

#### Core Temperature (Tc) Measurement

Body core temperature (Tc) was monitored using a thermonuclear probe (BW-TH1101, Billion, Shanghai, China) inserted 2 cm into the rectum.

#### Animal Preparation and Training

Before starting the heating and exercise protocols, the mice were acclimatized to the artificial climate chamber (ARQ-250, Shanghai GEMTOP Scientific Instrument), a motor-driven treadmill (BW-ZHPT-8-channel mice treadmill; Billion), and a rectal thermonuclear probe. Mice remained in their home cages and were subsequently placed in the chamber with a Ta of 27 ± 0.5°C and relative humidity of 55 ± 5% for 2 h each day for 2 weeks and were rested for 2 day before the start of the experiment. They had access to *ad libitum* food and water. Exercise acclimation was performed by making the mice run for 2 h each day for 2 weeks on a running wheel that rotated at a constant speed of 15 rpm/min. The rectal temperature of all mice was measured twice each day for 2 weeks before the experiment.

### Exploration of the Conditions to Be Used in the EHS Experiments

#### Environmental Conditions Were Selected Based on Survival Rate

All mice were weighed 1 h before the heat stroke experiment, and food and water were removed from the cages. To induce heat stroke, the mice were placed in an artificial climate chamber delivering one of four environmental conditions with relatively high temperature and humidity (37.5°C, 65% RH; 37.5°C, 70% RH; 39.5°C, 65% RH; and 39.5°C, 70% RH). During this process, the body temperature of each mouse rose gradually. The end point of heat stroke was when a mouse appeared exhausted or its Tc reached 41°C or greater, as used in previous studies (King et al., 2015, 2017). Following the onset of heat stroke, mice were immediately removed from the chamber, weighed, and returned to a Ta of 25°C with access to food and water *ad libitum*. We determined the survival rate of mice within 24 h after developing heat stroke and chose the conditions with a survival rate of close to 50% as the environmental conditions for the EHS study group. The Tc of each mouse was monitored at 10 min intervals for a further 24 h. The SHR group was rested at room temperature (23°C, 50% RH).

#### The Core Temperature of Heat Stroke Was Selected Based on Survival Rate

Mice were placed in the artificial climate chamber and were induced to heat stroke with a core temperature reaching 42, 42.5, and 43°C under conditions of 37.5°C, 65% RH. The survival rate of mice with heat stroke within 24 h was determined and the Tc with a survival rate of close to 50% was selected as the core temperature of heat stroke for the EHS group.

### EHS Experiment

Before starting the formal experiment, the trained mice were allowed 2 day of rest with free access to the running wheel within their cages. We chose the condition of 39.5°C, 65% RH for EHS modeling. As soon as the artificial climate chamber was calibrate to the target Ta (within ∼1 h), the chamber was opened, and the animal was quickly placed on the running wheel. The forced running wheel protocol was then initiated. Leon et al. used variable speed parameters during the EHS protocol as follows: initial running speed 2.5 m/min; this was increased 0.3 m/min every 10 min until the mouse reached a Tc of 41°C, which served as the threshold beyond which the running speed was kept constant (Department of Critical Care Medicine, 2016; King et al., 2017). In the current study, it is worth noting that the running wheel was kept at a constant speed of 15 rpm/min throughout the test. The end point of the EHS test was marked by signs of exhaustion in the mouse (defined as the mouse no longer keeping pace or unable to right themselves when placed on their backs) or a core temperature of 43°C.

At the end point, mice were removed from the climate chamber, weighed, and returned to their respective home cage. Tc continued to be monitored for 24 h into recovery or until death. The mice had free access to food and water. The 12 h light-dark cycle was maintained within the environment during the recovery period. The control group for EHS was the sham heat exercise (SHE) group. For this group, the running wheel rotated at a constant speed of 15 rpm/min, mice were forced to exercise for 1 h (including rest time) in the chamber with a Ta of 27°C and at 55% RH. Thus, the total running time was limited to 60 min (including rest periods) to ensure each mouse within a particular group had an equal amount of exercise. After the 60-min run, the treadmill was stopped, and mice were immediately removed from the chamber, weighed, and returned to a Ta of 25°C with access to food and water *ad libitum*.

### CHS Experiments

All animals were weighed 1 h before the heat stress experiment, and food and water were removed from the cages. To induce CHS, animals were placed in the artificial climate chamber. The CHS modeling condition was selected as 39.5°C, 65% RH, the same as for EHS modeling. During this process, the body temperature of each mouse gradually rose. The CHS end point was considered to be when the core temperature reached 43°C or exhaustion was apparent. At the onset of heat stroke, mice were immediately removed from the chamber, weighed, and returned to a Ta of 25°C with access to food and water *ad libitum*. Tc was monitored at 10 min intervals for a further 24 h. The control group for the CHS group was the sham heat rest (SHR) group, mice in the SHR group rested at room temperature (23°C, 50% RH).

### Weight Alteration

The body weight of mice was measured before heat stress and at the attainment of the Tc (43°C) or exhaustion. The changes in weight were calculated as follows: (weight before heat stress – weight at a Tc of 43°C or exhaustion)/weight before heat stress 100%.

### Plasma Biochemical Analysis

Blood samples were collected at different time points during recovery from heat stroke in mice. Samples were centrifuged at 3,000 rpm and 4°C for 30 min, and the supernatant was collected for further analysis. Assay kits were used to determine the levels of creatine kinase (CK; A032-1-1, Kanchenjunga Bioengineering, Nanjing, China); alanine transaminase (ALT; C009-2-1, Kanchenjunga Bioengineering); blood urea nitrogen (BUN; CO13-2-1, Kanchenjunga Bioengineering); myoglobin (MB; (CSB-E144004m, Cusabio); and interleukin 1 beta (IL-1β; CSB-E08054m, Cusabio).

### Histopathological Examination

Tissue specimens were fixed in 10% neutral buffered formalin, and then dehydrated and embedded in paraffin. Sections of 4 μm thickness were stained with hematoxylin and eosin (H&E). Kidney samples were analyzed by light microscopy, as described by Paller and Neumann (1991). In this system, under × 400 magnification, 10 fields of view with 100 renal tubules were randomly observed. The standard procedure was as follows: evident renal tubule expansion and flat cells (1 score); the appearance of tube-type cells in renal tubules (2 score); the appearance of some exfoliated necrotic cells in the lumen of the renal tubules but not tubular or cell fragments (1 score); vacuolar degeneration (1 score); and karyopyknosis. Masson’s staining revealed collagen fibers as blue in color. The degree of renal and gastrocnemius muscle interstitial fibrosis was observed in 10 randomly selected non-overlapping fields under an optical microscope. Semi-quantitative analysis was then performed using Image Pro Plus software (Bethesda, MD, United States), and the results were calculated as the percentage of interstitial collagen deposition in relation to the total area of the tubular interstitium.

### Evans Blue Dye Extravasation

Evans Blue dye (EBD; 2%, 4 ml/kg) was administered via the tail vein for 2 h. Under deep anesthesia, mice were sacrificed by cardiac perfusion. Then, the gastrocnemius muscle was immediately removed and separated. Whole gastrocnemius muscle samples were subsequently weighed and homogenized with 3 ml of 50% trichloroacetic acid, then centrifuged at 15,000 g for 30 min. The supernatant was mixed with an equal volume of trichloroacetic acid with ethanol. After overnight incubation (4°C), the samples were centrifuged again (15,000 *g*, 30 min) and supernatant was measured using a spectrofluorophotometer (excitation wavelength 620 nm and emission wavelength 680 nm).

### Imaging of Fluorescence

During the recovery time of 6 h, the gastrocnemius muscles of mice with heat stroke were snap-frozen in isopentane-cooled liquid nitrogen. Cryostat microtomy was performed to obtain 5-μm gastrocnemius muscle sections. Fluorescence imaging of EBD was performed using a filter set with a 510–560 nm band-excitation filter and a 590 nm long-pass emission filter.

### Transmission Electron Microscopy

Six hours after the onset of heat stroke, the mice were euthanized and the gastrocnemius tissues were washed with precooled PBS (pH 7.4). Part of the gastrocnemius tissue was then removed and incubated overnight in 0.1 M PBS (pH 7.4), containing 2.5% glutaraldehyde. The target tissues were cut into 50 μm-thick sections using a vibratome. Selected areas of the gastrocnemius were post-fixed in 1% osmium tetroxide for 1 h, dehydrated in a graded ethanol series, and embedded in epoxy resin. Polymerization was performed at 80°C for 24 h. Ultrathin sections (100 nm) were cut, stained with uranyl acetate and lead citrate, and viewed under a JEM2000EX transmission electron microscope (JEOL, Tokyo, Japan).

### Intravital 2-photon Microscopy

We injected a Fluo-4AM calcium ion fluorescent probe (S1056, Beyotime) into the tail vein of each mouse. Fluo-4AM, one of the most commonly used fluorescent probes for the detection of intracellular calcium concentration. After entering the cell, Fluo-4AM can be cleaved into Fluo-4 by intracellular esterase, thereby binding calcium ions to produce a strong green fluorescence.

Mice were anesthetized by injecting 60 mg/kg sodium pentobarbital into the abdomen. In addition, 100 μL of the 200 μM calcium ion probe and rhodamine B isothiocyanate-dextran, which labels blood vessels with red fluorescence (Sigma, R9379), were injected via the tail vein. The hair on the legs of mice was then shaven, and a small incision was made in the skin to expose the calf muscles. Mice were then placed on a warm safety pad in a holder. A suction cup was affixed to establish a negative pressure system. The suction cup was attached at the calf muscle, and 100 μL of physiological saline was added dropwise to the suction cup during the whole process. Green fluorescence signals were then observed and recorded using intravital 2-photon microscopy.

### Statistical Analysis

Statistical analysis was performed using GraphPad Prism 5.0 (GraphPad Prism Software, San Diego, CA, United States). Survival was expressed as a percentage. Data were presented as the median value, interquartile range, and minimum and maximum values. Multiple comparisons were performed by one-way analysis of variance (ANOVA). Bonferroni correction was conducted for *post hoc* comparisons, when warranted. At least three independent experiments were performed to confirm the results. Survival was analyzed using the Kaplan-Meier method and compared using the log-rank test. A two-tailed *P* ≤ 0.05 was considered to indicate statistical significance.

## Results

### Survival Rate of Mice Within 24 h of the Onset of Heat Stroke

**FIGURE 1:**
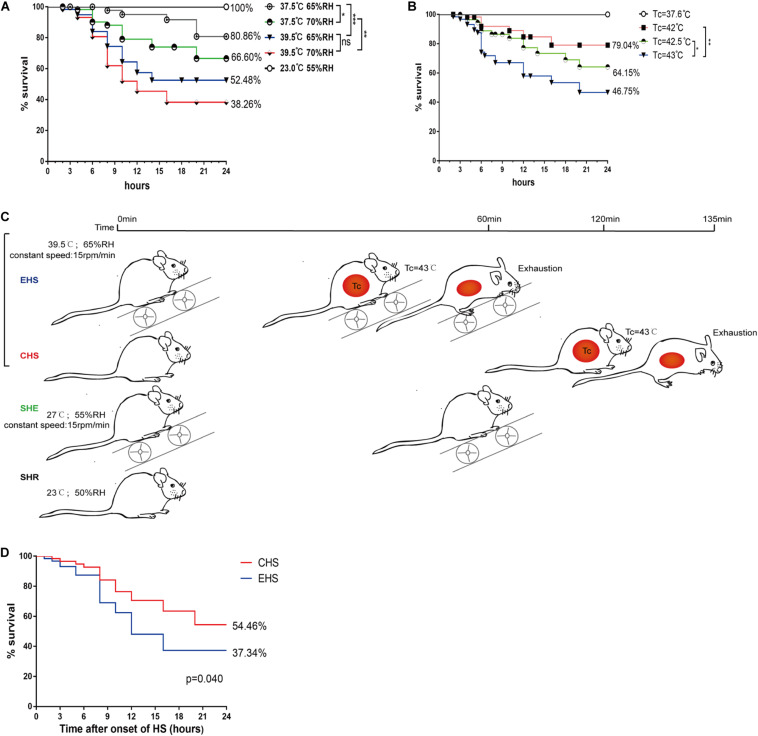
Survival rate of mice within 24 h of onset of various heat stroke conditions. **(A)** Survival rate of mice with heat stroke within 24 h under four different conditions of ambient temperature and relative humidity (RH) (*n* = 60 mice/group); **(B)** Survival rate of mice with three levels of high core temperatures within 24 h (*n* = 60 mice/group); **(C)** A timeline of exertional heat stroke (EHS) and classic heat stroke (CHS) protocols; **(D)** Survival rate of mice with CHS and EHS within 24 h following exposure to 39.5°C 65% RH (*n* = 60 mice/group). Survival was assessed by the Kaplan–Meier method. Significance was set at *P < 0.05, **P < 0.01, ***P < 0.001.

The survival rate of mice was closely related to the degree of tissue and organ damage. First, four sets of relatively high levels of temperature and humidity (37.5°C, 65% RH; 37.5°C, 70% RH; 39.5°C, 65% RH; and 39.5°C, 70% RH) were designed to make increase the susceptibility of mice to heat stroke based on the thermal index. A Tc of ≥41°C, or signs of exhaustion in a mouse, were considered the end point of heat stroke, as used in previous studies (King et al., 2017). Subsequently, mice with heat stroke were immediately transferred to a room-temperature environment where survival rates were determined for the four different conditions within 24 h. As shown in Figure 1A, the survival rate of mice was 52.48% under conditions of 39.5°C, 65% RH, which is similar to the heat stroke mortality rate in human patients. Next, the survival rate of mice was determined throughout the higher core temperatures (Tc of 42, 42.5, and 43°C) under conditions of 37.5°C, 65% RH. As expected, mice with a Tc that reached 43°C had a low survival rate of 46.75%, (Figure 1B). Finally, 39.5°C, 65% RH was selected as the environmental conditions for heat stroke, with a Tc of 43°C or exhaustion as the end point for the heat stroke experiment. Mice in the EHS group were made to exercise on a running wheel at a constant speed of 15 rmp/min. Mice in the sham heat exercise (SHE) group (the control group for EHS) ran in a room-temperature environment with the same exercise parameters (Figure 1C). Mice in the sham heat rest (SHR) group were used as the control group for the CHS group; results showed that mice in the EHS group had a lower survival rate than those in the CHS group (Figure 1D; EHS group 37.34% vs. CHS group 54.46%). These results indicate that a relatively higher ambient temperature and humidity, as well as higher core temperature during heat stroke, could greatly affect prognosis and may lead to more severe tissue and organ damage.

### Temperature Curve for Mice Within 24 h of the Onset of Heat Stroke, and Weight Loss During Heat Stroke

**FIGURE 2:**
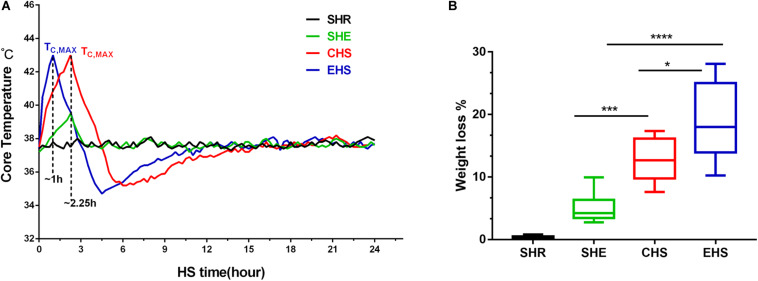
Temperature curve for mice with heat stroke within 24 h of onset of heat stroke and weight loss during heat stroke. **(A)** The core temperature (Tc, °C) of mice exposed to 39.5°C, 65% relative humidity (RH) was monitored at 10 min intervals; **(B)** Percent body weight loss from different groups after the onset of heat stroke, *P < 0.05, ***P < 0.01, ****P < 0.001; ANOVA least significant difference test. Data are presented as the median value, interquartile range (box), and minimum and maximum values.

Mice with heat stroke lost their ability to maintain thermoregulatory balance (DeMartini et al., 2014), as indicated by the changes in Tc within 24 h in each group. As shown in Figure 2A, mice with EHS reached a body temperature of 43°C within a shorter time period than those with CHS. After reaching the target Tc of heat stroke, the body temperature dropped rapidly and gradually returned to basal body temperature at approximately 6 h. This change was consistent with the three-phase change in body temperature that is characteristic of heat stroke. Mice running at room temperature for 60 min showed no signs of heat stroke, and their body temperature only showed a slight regulatory increase. Although mice with EHS were exposed to the experimental conditions for a shorter duration than those with CHS, the former underwent greater weight loss during heat stroke (Figure 2B).

### Sever Systemic Organ Damage in Mice With EHS

**FIGURE 3:**
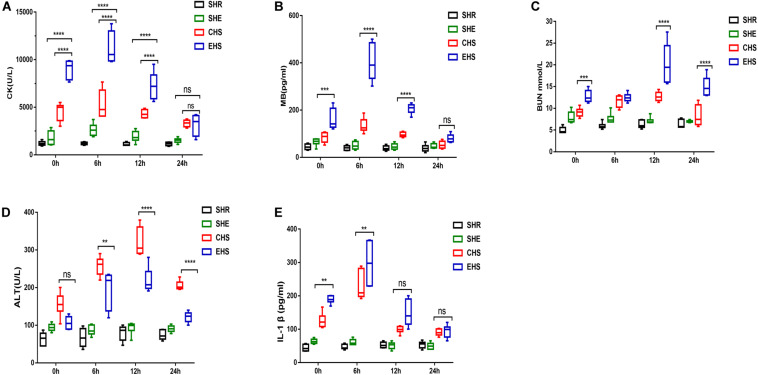
Severe systemic organ damage in mice with EHS **(A)** Analysis of serum levels of creatine kinase (CK) (*n* = 6 mice/group, ****p < 0.0001, ANOVA LSD test). **(B)** Analysis of serum levels of myoglobin (*n* = 6 mice/group, ****p < 0.0001, ***p = 0.0006, ANOVA LSD test). **(C)** Analysis of blood urea nitrogen (BUN) (*n* = 6 mice/group, ***p = 0.0001; ****p < 0.0001, ANOVA LSD test). **(D)** Analysis of serum alanine aminotransferase (ALT) (*n* = 6 mice/group, **p = 0.0054; ****p < 0.0001, ANOVA LSD test). **(E)** Analysis of serum interleukin 1 beta (IL-1β) (*n* = 6 mice/group,CHS VS EHS in 0 h, **p = 0.0033; CHS VS EHS in 6 h, **p = 0.0011, ANOVA LSD test). All data are presented as the median, interquartile range (box), and minimum and maximum values.

The survival rate of mice with EHS was lower than for those with CHS (Figure 1D). In addition, impaired histological structure and function were observed concurrently with abnormal serum indicators. Typical serum indicators including CK and MB (Warren et al., 2002; Casa et al., 2012) can be determined to specifically assess skeletal muscle injury; therefore, we measured the serum levels of these indicators from 0 to 24 h after the onset of heat stroke. As shown in Figures 3A,B, the results strongly supported the establishment of an EHS-induced RM animal model, as evidenced by the elevated serum levels of CK and MB. The CK level mostly exceeded 10,000 U/L at 6 h after EHS which was 10-fold that of the SHR group; however, there was only a slight increase in the CHS group. These findings suggest that skeletal muscle was severely damaged by EHS. Additionally, BUN reflects glomerular filtration rate and degree of renal injury. As shown in Figure 3C, the BUN level of mice with EHS was high from 12 to 24 h, indicating that these mice suffered from impaired renal function during the later stages of heat stroke. IL-1β levels are used as an indicator to quantify inflammatory injury (Dinarello, 1996; Dematte et al., 1998). In mice with EHS, IL-1β levels were significantly increased at 0 h, and reached a peaked at 6 h. Mice with CHS also showed a similar trend, but their IL-1β levels were lower than those of mice with EHS (Figure 3E). Therefore, although mice with heat stroke were removed from the high-temperature environment in a timely manner, they still exhibited an inflammatory reaction. The level of ALT in mice with CHS was higher than that in mice with EHS, although the trend was similar (Figure 3D). In summary, these results indicate that systemic organ damage in mice with EHS was more severe than that in mice with CHS.

### Severe Rhabdomyolysis Occurred in Mice With EHS

**FIGURE 4:**
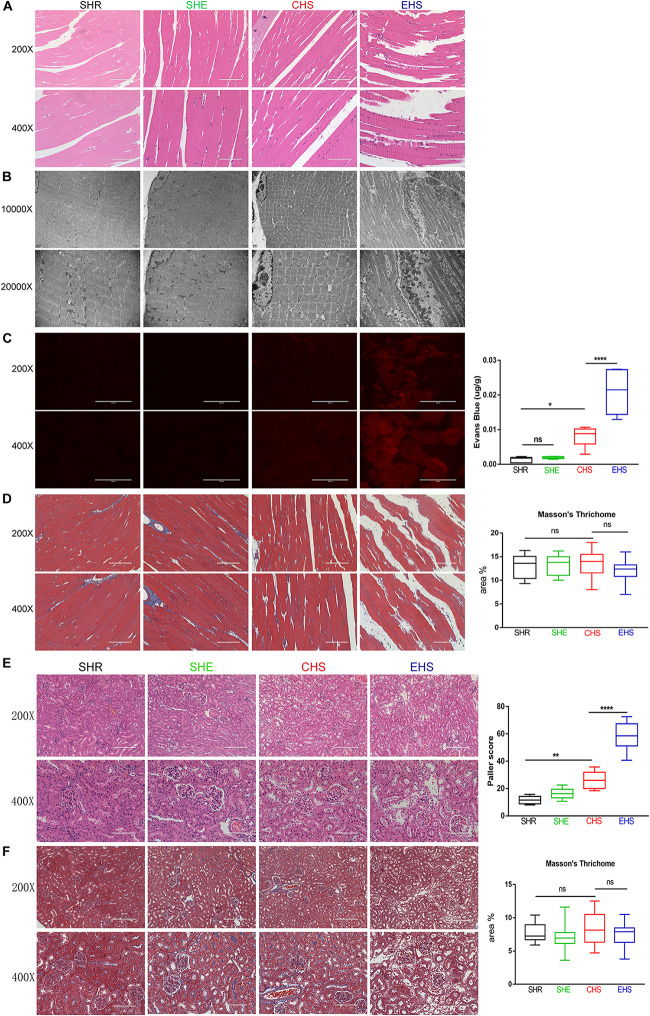
Histological examination of the skeletal muscle and kidney. **(A)** Histological examination of the skeletal muscle in mice by H&E staining. **(B)** Transmission electron microscopy was used to examine rhabdomyolysis in mice. **(C)** Evans Blue fluorescence and detection of Evans Blue penetration into the tissue. *n* = 6 mice/group; *p < 0.05, ****p < 0.0001. ANOVA LSD test. Data are presented as median value, interquartile range (box), and minimum and maximum of all data. **(D)** Representative images and quantitative analyses of Masson’s trichrome staining for gastrocnemius muscle tissue. **(E)** H&E stained images of the histopathological changes in renal tissue and Paller scores. *n* = 6 mice/group; **p = 0.0063, ****p < 0.0001. ANOVA LSD test. Data are presented as median value, interquartile range (box), and minimum and maximum of all data. **(F)** Representative images and quantitative analyses of Masson’s trichrome staining of renal tissue.

To evaluate histological skeletal muscle injury, gastrocnemius muscle tissue collected at 6 h after the onset of heat stroke was stained with H&E. As shown in Figure 4A, a deficiency in muscle fibers and myofibrillar sparsity and necrotic cells was observed in the EHS group compared with that in the SHR group. The gaps between muscle fibers in the EHS group were also found to be larger. However, the muscle fibers of mice with CHS showed no evident deficiency. Muscle fibers in the SHE group were histologically normal. Subsequently, further transmission electron microscopy was performed. As shown in Figure 4B, myolytic foci were found to be widespread in the gastrocnemius muscle of mice with EHS. Sarcomere damage, serious muscle dissolution, and fragmentary muscle fibers were seen in the EHS group. Light and dark bands of muscle cells became fuzzy and a thickened Z line was also observed in mice with EHS. In addition, more severe degradation of mitochondrial structure was evident in the EHS group. The myofibrils were arranged tidily, and the light and dark bands were arranged neatly; in addition, sarcomeres and the Z lines were clear in the CHS, SHE, and SHR groups.

Evans Blue dye is a permeable dye that can be easily taken up by degenerated or damaged cells, but not by cells with an intact membrane, and emits a bright red fluorescence upon excitation (Smith et al., 2015). Therefore, an EBD penetration experiment was conducted as described above. As shown in Figure 3C, the gastrocnemius muscles of mice with EHS emitted a strong red fluorescence; however, the red fluorescence in gastrocnemius muscle of mice with CHS was weak, and no red fluorescence was observed in the SHR and SHE groups. Accumulation of EBD was evident in the gastrocnemius muscles of mice with EHS, but only slight accumulation was noted in mice with CHS. These results indicated that the membrane integrity of muscle cells in mice with EHS was deficient. To determine whether substantial muscle cell damage caused tissue fibrosis in the short term after EHS, we further conducted Masson’s trichrome staining of the gastrocnemius muscles of mice in each group at a time point of 6 h after onset of heat stroke. Unexpectedly, interstitial fibrosis was not found in any of the groups (Figure 4D).

Acute kidney injury is a known complication of rhabdomyolysis. As shown in Figure 4E, staining of kidney tissue showed that the morphological structure of renal tissue was normal in the SHR and SHE groups. Renal tissue at 24 h after EHS showed the loss of brush borders, vacuolar degeneration, and necrosis in the epithelial cells, as well as dilatation, cast formation, and cell debris in the kidney tubules. Renal tissue from CHS mice also showed slight injury to renal tubules. Unexpectedly, no significant interstitial fibrosis in the kidneys was observed at 24 h in EHS group and CHS group.

### Calcium Overload in Mice With EHS-Induced Rhabdomyolysis

**FIGURE 5:**
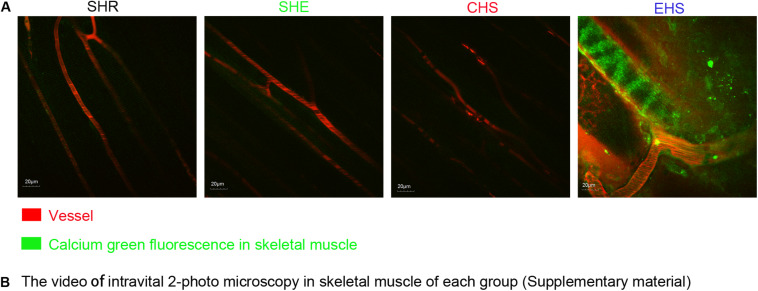
**(A)** Calcium ion fluorescence intensity of muscle tissue in four animal models observed by intravital 2-photo microscopy. An injection of 200 nM Fluo-4AM was administered to mice via the tail vein at 6 h after the onset of heat stroke. **(B)** The video of intravital 2-photo microscopy in skeletal muscle in each group. (As is shown in Supplementary Material, video SHR, video SHE, video CHS, video EHS).

Existing literature has reported that calcium overload often accompanies rhabdomyolysis along with EHS progression (Riazi et al., 2014). Therefore, we verified whether calcium overload occurred within the skeletal muscle of mice with EHS. Fluo-4AM was injected into the tail vein of mice. Using intravital 2-photon microscopy and 488 nm excitation waves, a strong green fluorescence signal was observed in the skeletal muscles of mice with EHS (Figure 5B; video EHS, as shown in the Supplementary Material); however, the muscles of mice in other groups did not yield a similar signal.

## Discussion

Exertional heat stroke is a critical emergency characterized by a greater severity of symptoms, more rapid development, and a higher morbidity and mortality (Zeller et al., 2011; Casa et al., 2012; Leon and Bouchama, 2015). During the period 2005–2009, the mortality rate of EHS in sports events in the United States remained high (Casa et al., 2012), and despite rapid cooling and active supportive treatment, 75% of heat stroke patients still developed multiple organ dysfunction syndrome (Leon, 2004). RM is an important complication in EHS patients, and their CK value can rise above 5,000 U/L (Tseng et al., 2016); mortality rates of up to 50% are recorded (Levi et al., 2009; Welc et al., 2013). However, under experimental conditions, RM incidence and mortality rate are rarely low, which are clearly inconsistent with the clinical statistical data for EHS. In addition, CK levels have not been observed to increase significantly, and the detection of skeletal muscle injury is yet to be performed. Therefore, in the current study, we set out to develop a modified animal model for EHS to better simulate the clinical characteristics of patients with EHS.

Mortality rates due to heat stroke, as reported in existing literature, are inconsistent but are mostly reported to be about 50%. In the current study, we designed four EHS conditions with relatively high levels of temperature and humidity (37.5°C, 65% RH; 37.5°C, 70% RH; 39.5°C, 65% RH; and 39.5°C, 70% RH). We determined the survival rate within 24 h, and chose conditions with an approximate 50% survival rate for EHS modeling. We then defined heat stroke as a Tc reaching 42, 42.5, and 43°C under the conditions of 37.5°C 65% RH, respectively, and found that mice with a 43°C Tc had an almost 50% mortality rate. We therefore chose the condition of 39.5°C 65% RH for EHS modeling. The end point of EHS evaluation was exhaustion or core temperature reaching 43°C. Finally, the 24 h survival rate of the EHS group under these conditions was 37.34% (Figure 1D); therefore, the high mortality of the EHS group was consistent with characteristics observed in EHS patients.

Exertional heat stroke patients are mostly young adults who exercise vigorously over an extended duration in high temperatures and high humidity levels (Casa et al., 2012). Therefore, to represent typical EHS patients, we selected mice at sexual maturity from 8 to 9 weeks of age. After 2 weeks of adaptive training, mice were formally tested at 10–11 weeks. At this age, mice have a better grasping and crawling ability, which might be in line with the prevalence of EHS in young and middle-aged people. However, Leon et al. (King et al., 2015, 2017) used relatively older mice, mice in this age (4-month-old) might have impaired body temperature thermoregulation and immune regulation. The experimental conditions of 39.5°C 65% RH are considered to be high levels of temperature and humidity, which is consistent with the environmental factors encountered by EHS patients.

In humans, a serum CK level over 5,000 U/L (five times the upper normal limit of 1,000 U/L) warrants a diagnosis of rhabdomyolysis (normal range is 10∼205 U/L) (Furman, 2015; Cervellin et al., 2017). In our model, the CK level in mice with EHS mostly exceeded 10,000 U/L at 6 h in the recovery period (Figure 3A; normal range was 800–1000 U/L tested by us). Although the physiological parameters of mice and humans are different, the specific CK values (>10,000 U/L) and the ratio of the CK values between the control group and EHS group were in line with the diagnostic criteria for human RM. H&E staining, EBD penetration, and transmission electron microscopy confirmed that the gastrocnemius muscles in the EHS group were severely damaged (Figures 4A–C). Although there was a large amount of cell death in gastrocnemius muscle, we did not observe evident fibrosis in muscle interstitium, which indicated that the underlying pathological process in the skeletal muscle following the occurrence of EHS is always happened on local tissue injury rather than fibrosis. Similarly, renal tubular epithelial cells showed significant levels of necrosis at 24 h after EHS, but no fibrosis. The possible reason for the severe cell death in skeletal muscle and renal tubules without fibrosis was that the EHS mice remained at a stage of stress damage and was associated with EHS disease characteristics. Tissue injury, rather than fibrosis, may be the earliest pathological feature of EHS. These results suggest that EHS is an acute, rather than a chronic, progressive disease with a very limited effective treatment window. A growing number of studies suggest that heat stroke is actually a systemic inflammatory response syndrome, (Bouchama and Knochel, 2002). Interleukin-1 was the first known mediator of systemic inflammation induced by strenuous exercise (Cannon and Kluger, 1983). A significant increase was detected in IL-1β levels in the serum of EHS or CHS mice at the onset of heat stroke; furthermore, its levels peaked at 6 h, indicating that acute inflammation may occur during the early stages of heat stroke. This could be due to the direct toxic effects of heat on the cells, as well as microcirculatory disorders, which result in damage to tissues and organs. These damaged structures release damage-associated molecular patterns (DAMPs) molecules, including HMGB1, to promote local aseptic inflammation and changes in intestinal permeability.

In addition, existing literature reports that calcium overload often accompanies the incidence of RM along with EHS progression (Riazi et al., 2014). Therefore, we further investigated calcium overload in skeletal muscles of the EHS group through the use of real-time intravital 2-photon microscopy. The results showed that no calcium overload occurred in the CHS, SHE, and SHR groups (Figure 5B, as shown in the Supplementary Material); these groups rarely develop RM, indicating that the calcium overload might be the main cause of RM induced by EHS. This hypothesis is also supported by existing literature (Carter et al., 2005). We suggest that our model might be suitable for studying the mechanism of EHS-induced RM.

## Conclusion

We constructed an EHS-induced RM animal model which had similar characteristic to EHS patients. This model will facilitate research into the pathogenesis of EHS-induced RM.

## Data Availability Statement

All datasets generated for this study are included in the article/Supplementary Material.

## Ethics Statement

The animal study was reviewed and approved by Animal care and use Committee of the Southern Medical University.

## Author Contributions

S-XH performed most of the experiments and contributed to the design of the study, to the interpretation of data, and to the writing of the manuscript. RL and H-HY contributed to the collection of data shown in Figure 1. S-XH and RL also performed the intravital 2-photon microscopy experiment, and analyzed the data. Z-QW, Y-MP, and J-HH contributed to the interpretation of data. QM supervised the study.

## Conflict of Interest

The authors declare that the research was conducted in the absence of any commercial or financial relationships that could be construed as a potential conflict of interest.
